# The Four Core Genotypes mouse model: evaluating the impact of a recently discovered translocation

**DOI:** 10.1186/s13293-024-00665-5

**Published:** 2024-10-31

**Authors:** Carrie B. Wiese, Barbara Soliman, Karen Reue

**Affiliations:** Department of Human Genetics, David Geffen School of Medicine, University of California, Los Angeles, CA 90095 USA

**Keywords:** Sex differences, X chromosome, Y chromosome, XY* mouse model, Gene expression

## Abstract

The Four Core Genotypes (FCG) mouse model has become a valuable model to study the mechanistic basis for biological sex differences. This model allows discrimination between influences of gonadal sex (ovaries or testes) from those associated with genetic sex (presence of XX or XY chromosome complement). FCG mice have illuminated distinct effects of gonadal and chromosomal sex on traits ranging from brain structure and behavior to vulnerability to obesity, atherosclerosis, multiple sclerosis, Alzheimer’s and other diseases. A recent study determined that the Y^*Sry*−^ chromosome used in a specific line of C57BL/6J FCG mice harbors nine genes that have been duplicated from the X chromosome. This report raised concern that scores of publications that previously used the FCG model may therefore be flawed, but did not provide details regarding how studies can be evaluated for potential impact (or lack of impact) of the translocation. Here we (1) provide a practical description of the genetic translocation for researchers using the FCG model, (2) document that a majority of the studies cited in the recent report are unlikely to be affected by the translocation, (3) provide a scheme for interpreting data from studies with FCG mice harboring the Y^*Sry*−^ translocation, and (4) delineate expression levels of the nine translocated genes across tissue/cell types as a filter for evaluating their potential involvement in specific phenotypes.

**Supplementary Information:**

The online version contains supplementary material available at 10.1186/s13293-024-00665-5.

## Background

Animal models, particularly mouse models, are widely used to investigate the role of biological sex in both health and disease. The recent emphasis on understanding how biological sex influences physiology has led to increased inclusion of both male and female mice in preclinical studies, and the inclusion of sex as a biological variable in preclinical and clinical research is now expected by major biomedical research funding agencies in the United States, Canada, and the European Union [1].

Biological sex factors include both genetic sex (XX or XY chromosomes) and gonadal sex (the presence of ovaries or testes) (2). A delineation of how each of these factors contributes to sex differences is highly relevant to human health and disease. The levels of gonadal hormones are dynamic during the lifetime, with low levels prior to puberty and after midlife. On the other hand, the genes on the sex chromosomes are potentially active in cells throughout the body across the lifespan. A determination of how each of these factors impacts disease at different life stages may illuminate the mechanisms by which one sex is more vulnerable or more protected from specific conditions [3, 4].

As the appreciation for the importance of both chromosomal and gonadal sex determinants has grown, investigators have turned to experimental models that allow the discrimination of these two factors. The most widely used model of this type is the Four Core Genotypes (FCG) mouse model, and the origin and application of this model has been described in detail in excellent reviews [2, 5]. This model allows the development of mice with four genotypes within a single mouse litter: XX chromosomes with ovaries, XX with testes, XY with ovaries, and XY with testes. The decoupling of chromosomal and gonadal type in this model derives from a Y chromosome that lacks the *Sry* gene due to a spontaneous deletion (referred to herein as Y^*Sry*−^), combined with an *Sry* transgene on chromosome 3, such that testis development is not determined by the Y chromosome. When the four genotypes are compared in a 2 × 2 array, it is possible to determine whether a trait is influenced by gonadal type, sex chromosome complement, or an interaction between the two. These distinct aspects of biological sex cannot be parsed using standard male and female organisms. To date, scores of publications have utilized FCG mouse models to identify mechanisms underlying sex differences in traits ranging from brain structure and behavior to development of obesity, atherosclerosis, multiple sclerosis, Alzheimer’s and other diseases ([2]; Supplementary Table 1).

Recently, it was reported that the Y^*Sry*−^ chromosome in one FCG mouse strain contains nine genes that have been duplicated and translocated from the X chromosome [6]. The article included a non-curated list of 98 publications using FCG mice (Supplemental Table 1 in [6]) that may raise concerns that those studies are flawed without evaluating whether the Y^*Sry*−^ translocation is likely to impact the conclusions from those publications. Here we aim: (1) to provide a practical description of the genetic translocation for researchers using the FCG model, (2) to document that a majority of the FCG studies cited in the Panten et al. report are supported by additional information that reduces the likelihood of erroneous conclusions attributable to the Y^*Sry*−^ translocation, (3) to provide a scheme for interpreting data from studies with FCG mice considering the Y^*Sry*−^ translocation, and (4) to assess relative expression levels of the nine translocated genes across tissue/cell types as a filter for evaluating their potential involvement in specific phenotypes.

### Genetic translocation identified on the Y^*Sry*−^ chromosome in a C57BL/6J Four Core Genotypes model

Panten et al. [6] performed single cell RNA-sequencing in liver and spleen from C57BL/6J FCG mice and observed higher expression levels (~twofold) for a set of X chromosome genes in XY^*Sry*−^ compared to XX mice. This was unexpected given that most genes on the X chromosome are expressed at similar levels between XX and XY cells due to X chromosome inactivation, or at higher levels in XX cells due to escape of specific genes from X chromosome inactivation (3–7% of X chromosome genes in the mouse) [7]. Panten et al. therefore investigated the basis for higher expression of some X chromosome genes in XY^*Sry*−^ compared to XX cells and determined (using DNA sequencing and DNA fluorescence in situ hybridization) that a 3.2 MB portion of the X chromosome had been duplicated and inserted into the Y^*Sry*−^ chromosome. The duplicated/translocated region originated from a segment that is adjacent to the X chromosome pseudoautosomal region, which may be subject to increased rates of genetic exchange between X and Y chromosomes [8]. The practical result of the translocation in this FCG strain is that its Y^*Sry*−^ chromosome harbors nine protein coding genes that are normally present only on the X chromosome (described in detail in a subsequent section). The dosage of the vast majority of the ~1000 X chromosome genes are not affected by this alteration, and Panten et al. also demonstrate that the duplication/translocation does not significantly impact autosomal gene expression [6].

During the past several years, the Y^*Sry*−^ chromosome has been bred onto different genetic backgrounds. Many of the early studies with FCG mice were performed on an outbred MF1 genetic background, which Panten et al. demonstrated does not harbor the translocation [6]. To generate an inbred strain to enhance utility for some types of biomedical research, the MF1 Y^*Sry*−^ chromosome was subsequently backcrossed to the inbred C57BL/6J strain through several generations. This C57BL/6J FCG strain was distributed to several investigators, and in 2010 was also deposited at the Jackson Laboratory (Jackson Laboratory strain 010905). Using frozen DNA samples from different stages of this process, it was determined that the MF1 strain does not carry the translocation, and that it occurred as a chance event during backcrossing from MF1 to C57BL/6J. The MF1 strain was also used by independent investigators to develop independent FCG strains on other genetic backgrounds (CD1, SJL). Having the FCG model on an inbred C57BL/6J background is valuable, and fortunately, a C57BL/6J FCG line that does not carry the translocation has been independently developed [6] and has recently been made available to the biomedical research community (Jackson Laboratory strain 039108).

### What is the impact of the C57BL/6J Y^*Sry*−^ translocation on studies previously performed with the FCG model?

The FCG models have been utilized by numerous investigators over the past two decades. Panten et al. provide a table in their publication (Supplementary Table 1 in [6]) that lists 98 studies performed with FCG mice that does not include annotation or context that allows the reader to evaluate whether or how the newly discovered translocation on the Y^*Sry*−^ chromosome might alter the interpretation of results. In practice, studies with FCG mouse models are seldom performed in a vacuum, but rather are combined with other studies to corroborate and extend the findings. Often, studies with FCG mice are undertaken after observing sex differences in humans or wild-type animals. The FCG model is then enlisted to reveal the relative contributions of gonadal sex and chromosomal sex. Results from FCG studies then ideally serve as a gateway to further investigation of either gonadal or chromosomal sex determinants [3, 5, 9]. When effects of gonadal sex are identified, subsequent studies may include the manipulation of acute gonadal hormone levels using techniques such as surgical gonad removal, hormone replacement, and chemical or genetic inhibition of gonadal hormone action. When chromosomal sex is implicated in FCG studies, further studies may assess the role of X and Y chromosome dosage with models such as XY* (discussed below). Thus, the results obtained in many studies with FCG models are often supported by several additional lines of evidence.

**Fig. 1:**
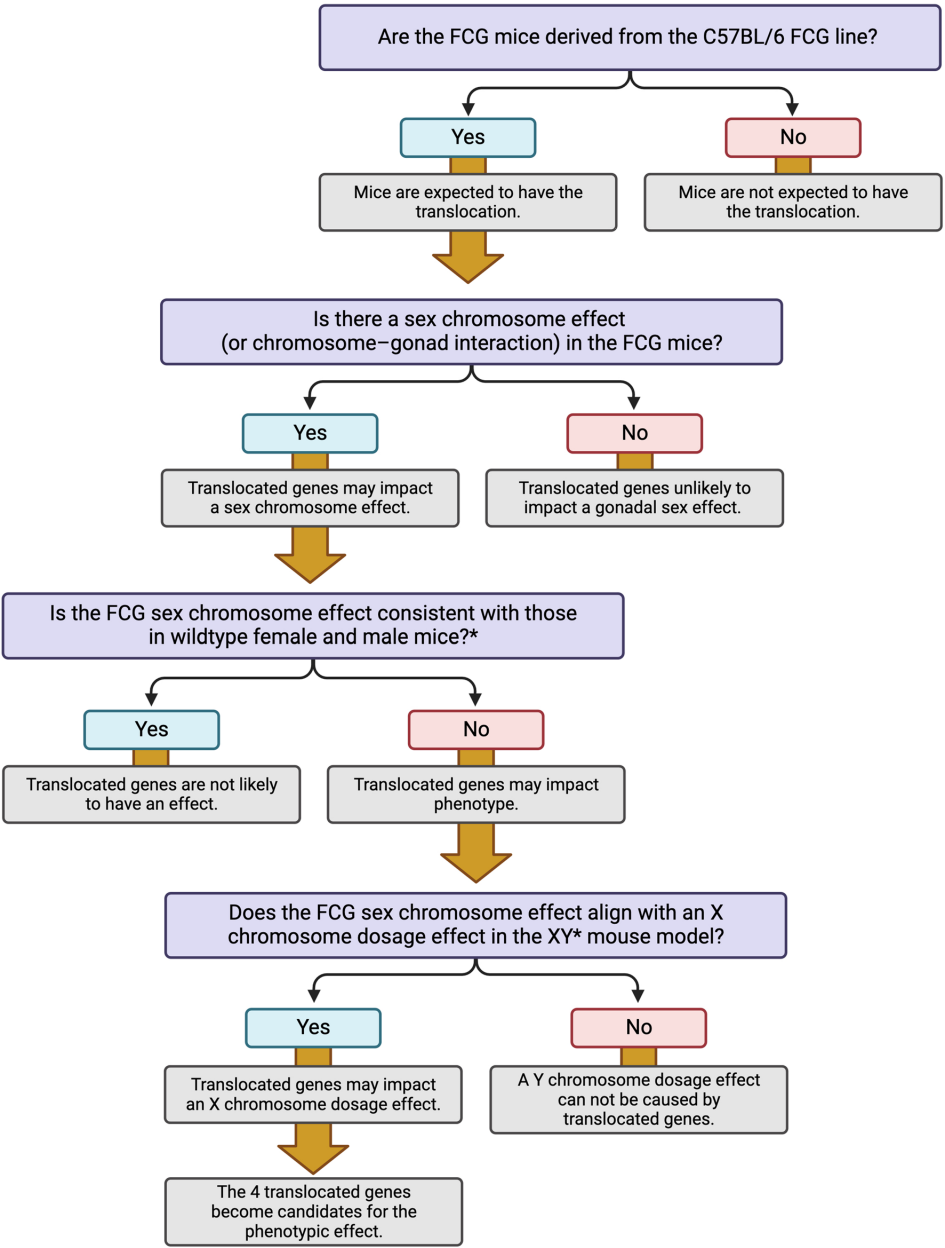
Flow diagram to evaluate potential influence of Y^*Sry*−^ chromosome translocation in existing studies performed with Four Core Genotypes mice. Decision tree used to evaluate published FCG mouse studies cited in Panten et al. for possible influence by the Y^*Sry*−^ chromosome translocation. Using this logic tree and additional published data, we identified studies shown in Supplementary Table 1 as likely exempt from complications that could arise from the translocation. *FCG XX vs. XY mice show same relationship as wild-type female (XX with ovaries) vs. male (XY with testes) mice. Figure was created with BioRender.com released under a Creative Commons Attribution-NonCommercial-NoDerivs 4.0 International License

We propose that existing publications related to studies in FCG mice (e.g., from Supplementary Table 1 in [6]) must be examined carefully to determine whether the results could be influenced by the Y^*Sry*−^ translocation. We have previously presented general schema for the analysis of sex determinants using the FCG mouse model [3, 4, 9]. Here, we present a flow chart that can be used to evaluate whether results from previous studies with FCG mice may be influenced by the presence of the translocation of X chromosome genes to the Y^*Sry*−^ chromosome (Fig. 1). Using the logic tree in Fig. 1 to evaluate studies that are cited in Supplementary Table 1 from Panten et al., we found that the majority of those studies are unlikely to be affected by the translocation, and we annotate the reasons for this in Supplementary Table 1. These include studies that were performed in MF1 FCG mice (which do not harbor the translocation), studies that identified only gonadal sex effects, studies that used additional mouse models to assess sex chromosome effects observed in FCG mice, and studies that identified specific sex chromosome genes that influence the trait. It is notable that despite the translocation, the C57BL/6J FCG model has provided critical data that has led to the identification of specific X and Y chromosome genes that influence neurological, autoimmune, cardiovascular, and metabolic diseases [10–15]. It is difficult to imagine how this would have occurred without valuable tools such as the FCG model.

### A practical guide to X and Y chromosome gene dosage in the C57BL/6J FCG and XY* strains

**Fig. 2:**
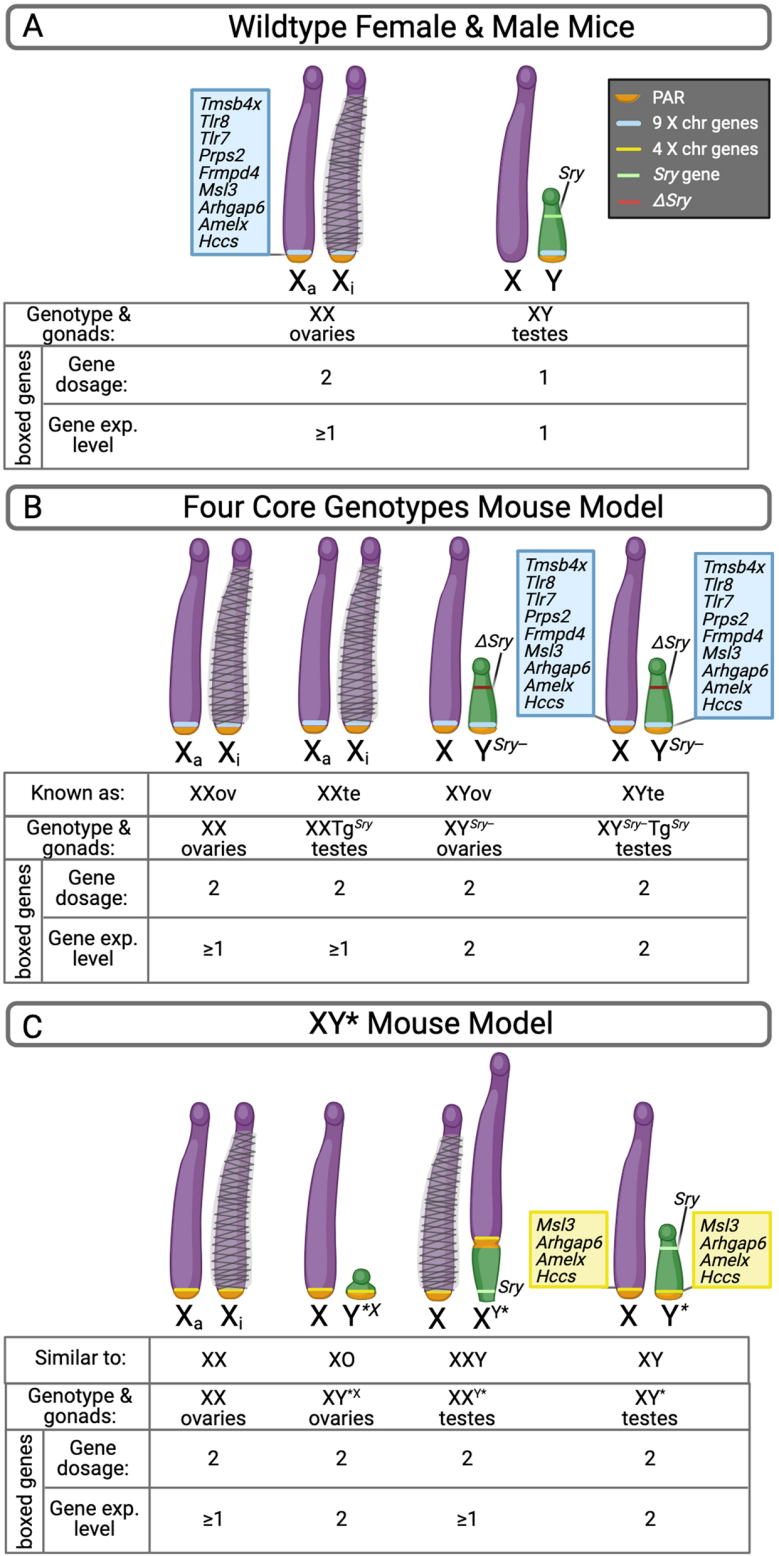
Chromosomal gene dosage and relative expression levels of X chromosome genes translocated to a C57BL/6J Y^*Sry*−^ chromosome. Schemes show sex chromosome and gonadal type in **A** wildtype female and male mice, **B** C57BL/6J Four Core Genotypes mice that carry the Y^*Sry*−^ translocation, and **C** XY* mice. X chromosome genes that are duplicated on the Y^*Sry*−^ chromosome (FCG model) or the Y* chromosome (XY* model) are shown in blue and yellow boxes, respectively. For each mouse model, the diagram shows sex chromosome and gonadal composition, the X and Y dosage, and expected gene expression levels for the X chromosome genes that are duplicated on the Y chromosome. The designation of “≥” under genotypes with two X chromosomes refers to potential expression of some boxed genes from both X alleles due to escape from X inactivation in specific cell types, which is known to occur for *Tlr7* and *Tlr8* (and potentially other boxed genes). Full names of the genes are provided in Fig. 3. PAR, pseudoautosomal region; *ΔSry*, deletion of *Sry* gene; Xa, active X chromosome; Xi, inactive X chromosome; XXov, XX chromosomes with ovaries, XXte, XX chromosomes with testes; XYov, XY chromosomes with ovaries; XYte, XY chromosomes with testes; Y^*Sry*−^, Y chromosome lacking *Sry* and containing translocation of 9 genes from the X chromosome; Tg^*Sry*^, *Sry* contained on a transgene within an autosome (not depicted in the diagram); Y*, Y^*X^, and X^Y*^ represent variants of the Y chromosome that occur in the XY* model and have the genetic composition illustrated in the diagram. Figure was created with BioRender.com released under a Creative Commons Attribution-NonCommercial-NoDerivs 4.0 International License

Here we suggest a scheme that may be employed to visualize the gene dosage on X and Y chromosomes in FCG and related mouse models, including the X genes that were translocated to the Y^*Sry*−^ chromosome (Fig. 2).

#### Wild-type female and male X and Y gene dosage and expression

In wild-type female and male mammals, females carry two alleles for X chromosome genes compared to one allele in XY males (Fig. 2A). The inactivation of one X in XX cells (Xi, X inactive) leads to similar expression levels for the vast majority of X chromosome genes in XX and XY cells. One exception is the small proportion of genes that escape X inactivation, which have higher expression levels in XX compared to XY cells; the degree of escape must be determined experimentally and differs by gene, cell type, age, disease status, and potentially environment and other factors [7, 16]. Only males express genes from the Y chromosome, which encodes ~80 protein coding genes. These include several genes involved in male reproduction and some orthologs of X chromosome genes that have diverged after specialization of the sex chromosomes occurred and X–Y recombination in non-pseudoautosomal regions ceased [17].

#### C57BL/6J FCG mouse X and Y gene dosage and expression

The FCG model is typically employed after sex differences are observed in standard female and male mice to interrogate the mechanism. The FCG sex chromosomes, including the Y^*Sry*−^ translocation characterized by Panten et al. [6], are represented in Fig. 2B. In the relevant C57BL/6J line of FCG mice, nine genes that reside on the X chromosome adjacent to the pseudoautosomal region are duplicated on the Y chromosome. This results in a copy of these genes on each type of X and Y chromosome present in the FCG animals (i.e., 2 genomic copies for each genotype, Fig. 2B). However, due to X inactivation, most of these genes are likely expressed at lower levels in XX (expressed from 1 allele) compared to XY genotypes (expressed from 2 alleles).

If results from FCG mice show a difference between mice with ovaries and those with testes, the interpretation is not likely to be affected by the translocation. A potential exception to this is cases in which there is an interaction between gonadal and chromosomal sex. If a trait is influenced by XX or XY chromosome complement, it may be due to: (a) a difference in the dosage of X or Y chromosome genes as occurs in wild-type mice, or (b) a higher expression level of one/some of the nine genes translocated to the Y^*Sry*−^ chromosome. However, additional clues may rule out condition (b). In particular, it is unlikely that the translocated genes are responsible for a difference in FCG mice if there is an FCG sex chromosome effect that is consistent with sex chromosomes in wild-type mice [for example, FCG XX vs. FCG XY mice show the same relationship as wild-type female (XX with ovaries) vs. wild-type male (XY with testes) mice]. This is because wild-type XY male mice do not have the translocated genes on the Y chromosome, so those genes are unlikely to be the reason for the segregation of the traits by XX and XY chromosome in FCG mice. Additionally, prioritizing potential candidates from the nine duplicated genes can be performed by considering the relevance of their cell type expression with the phenotype of interest (discussed in a subsequent section).

Panten et al. [6] made special mention of the *Tlr7* (toll-like receptor 7) gene in the Y^*Sry*−^ translocation, apparently because this gene has been implicated in autoimmune diseases such as lupus, which show a female bias. Of the nine genes translocated to the Y^*Sry*−^ chromosome, it is noteworthy that *Tlr7* and *Tlr8* both escape from X chromosome inactivation in specific immune cells that have roles in autoimmunity (B cells, monocytes, and plasmacytoid dendritic cell populations) [18, 19]. As such, these genes may be expressed from both the active and inactive X chromosomes in some immune cells of XX mice, thus reducing the dosage difference between XX and XY^*Sry−*^* cells*. The end result might be to make it less likely that *Tlr7* or *Tlr8* underlie XX vs. XY differences detected in C57BL/6 FCG mice. Other genes within the translocation may escape X inactivation in specific cell types, though this has not been well established.

#### XY* mouse model X and Y gene dosage and expression

Many investigators who detect sex chromosome effects in FCG mice perform follow-up studies to determine whether the number of X or number of Y chromosomes is likely to be the determinant. This is valuable as it also points towards specific candidate genes on either the X or Y chromosome. The XY* model contains a Y chromosome (known as Y*) with a duplication of part of the pseudoautosomal region; this allows unusual recombination events with the X chromosome to generate mice with nearly the equivalent of XX and XO genotypes (with ovaries) and XY and XXY genotypes (with testes) [2, 20] (Fig. 2C).

If a trait analyzed in XY* mice is influenced by Y chromosome dosage, animals with a Y (XY and XXY) will differ from those lacking a Y chromosome (XX and XO). If a trait is influenced by X chromosome dosage, animals with two X chromosomes (XX and XXY) will differ from those with one X (XY and XO). There is a slight complication in interpreting the latter results because four X chromosome genes (*Msl3*, *Arhgap6*, *Amelx*, and *Hccs*) are included in the pseudoautosomal duplication of the Y* chromosome (3). This results in a copy of these genes on each type of X and Y chromosome in XY* animals (i.e., 2 genomic copies for each genotype, Fig. 2C). However, due to X inactivation, the expression of these genes may be lower in XX and XXY genotypes (expressed from 1 allele) than in XO and XY genotypes (expressed from 2 alleles). Thus, if X chromosome dosage is implicated, the potential involvement of these four genes should be considered. Additional clues about whether any of the four genes are likely candidates for a particular trait can be derived by assessing the tissue/cell types that express these genes (discussed in a subsequent section).

A key value of identifying whether X or Y chromosome dosage is critical for a sex difference is that it suggests candidate genes on either the X or Y that may be associated with the trait of interest. If X chromosome dosage is implicated, some likely candidates are genes that exhibit differential expression levels in XX compared to XY cells, such as genes that escape X inactivation. Conversely, if studies from the XY* model implicate Y chromosome dosage, then Y-specific genes are likely candidates [17]. As indicated in Supplementary Table 1, multiple studies that used FCG mice and/or XY* mice to identify a role for X chromosome dosage subsequently progressed to identify a specific X chromosome gene with differential expression levels in females compared to males that influences the trait. These include the prominent X escape genes *Kdm6a* (Alzheimer’s disease resilience, multiple sclerosis, viral infection outcomes) and *Kdm5c* (obesity, vulnerability to drug adverse effects) [10–12, 14, 15]. As verified in these studies, both of these genes escape X inactivation in a wide array of tissues, and both encode epigenetic regulators (histone demethylases) that influence chromatin accessibility and expression of numerous genes. Importantly, these genes also escape X inactivation in humans, and their association with similar phenotypes in human cells and tissues has been demonstrated in some of the studies [11, 12, 15].

### Cell and tissue expression of nine X chromosome genes present on the Y^Sry−^ chromosome

**Fig. 3:**
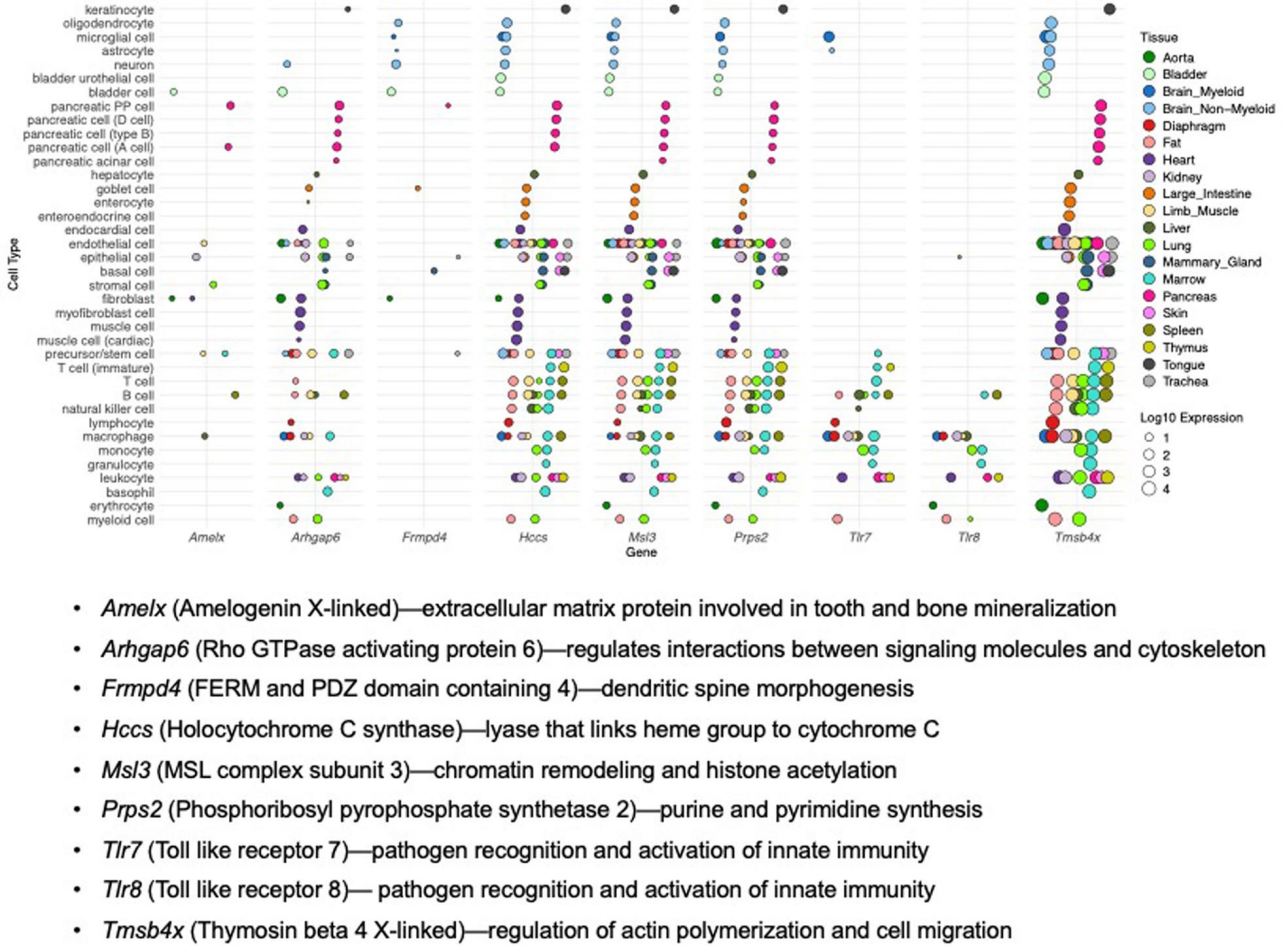
Tissue-type expression of nine *X chromosome genes present on the Y*^*Sry−*^* chromosome*. Single-cell RNA-seq data from FACS sorted cells of 20 tissue types were downloaded from the Tabula Muris Consortium [21]. Data were pooled from 3 month-old C57BL/6JN male and female mice. Gene expression values for the nine genes contained in the Y^*Sry*−^ translocation were normalized for sequencing depth per cell, and the average expression across each cell type within a tissue was calculated. A low expression cutoff of <1 cpm/cell was applied. Values from redundant cell types within a single tissue type (i.e., same cell type name but different clusters) were averaged and are represented as a single value for the specified cell type. A plot of log_10_(normalized expression) per cell type across tissues was generated with ggplot2 in R. Gene names and function are denoted beneath the plot

As delineated above and in Fig. 2, there are some circumstances in which the presence of the Y^*Sry*−^ translocation could implicate nine X chromosome genes as contributors to the difference between XX and XY mice. In these cases, an additional consideration is whether the gene is likely to influence the phenotype of interest based on its tissue pattern of expression. An analysis of single cell gene expression data from *Tabula Muris* [21] reveals that some of the genes have very low or limited tissue expression patterns (e.g., *Amelx*, *Frmpd4*; Fig. 3). These genes would be relevant candidates only for phenotypes involving specific cell types in specialized tissues. As expected, *Tlr7* and *Tlr8* are only expressed in specific immune cell populations; as described above, these genes also escape X inactivation in some immune cells, which might make them less likely to be responsible for differences observed between XX and XY FCG mice that carry the Y^*Sry*−^ translocation. The most widely and strongly expressed gene is *Tmsb4x*, which appears to be expressed at similar levels across numerous tissues. Thus, the involvement of genes on the translocated Y^*Sry*−^ chromosome as candidates for a sex chromosome effect can be prioritized based on whether they are expressed in a tissue pattern that corresponds with the phenotype.

## Perspective and significance

Panten et al. [6] have presented an important new piece of information regarding the genomic sequence of the Y^*Sry*−^ chromosome in a specific C57BL/6J FCG mouse strain. Animals with this chromosome have been used by researchers across fields for the past two decades. As sometimes happens with the availability of new technology that was not routine when the FCG model was developed (in this case, RNA-sequencing and long-read genome sequencing), new details are revealed. Here, we have sought to provide clarity and transparency regarding the potential impact of this finding on studies that have used the FCG model.

As we illustrate in Supplementary Table 1, numerous studies that have identified sex chromosome effects using the FCG model augmented those findings with additional analyses, in some cases narrowing down effects to a single gene using mice with specific gene knockout alleles. Through these methods, many of the studies have ruled out artifactual findings that could result from the presence of the nine gene translocation. Furthermore, a number of additional studies cited by Panten et al. were performed with FCG mice on non-C57BL/6J genetic backgrounds (primarily MF1) that do not carry the translocation. Thus, while it is critical for investigators using these mouse models to be aware of the new findings by Panten et al., it is also critical that data from previous studies be evaluated on a case-by-case basis and taking into account all evidence supporting their conclusions.

Moving to the future, as Panten et al. indicate, it will be possible to obtain a C57BL/6J FCG strain that has been verified to have the expected and desired genome sequence from a commercial vendor [6]. The FCG model has been extremely valuable in revealing significant roles for sex chromosome dosage in numerous physiological processes that extend far beyond their role as determinants of testis/ovary development. Given the demonstrated predisposition for recombination in regions of the sex chromosomes near the pseudoautosomal regions, it is recommended that routine genetic monitoring of FCG (and XY*) strains be performed, as is currently done with inbred strains at leading mouse vendors [22]. With conscious attention to rigorous maintenance and independent confirmation of findings, the FCG model will continue to be a valuable tool to illuminate roles for sex chromosome dosage in numerous physiological processes.

## Supplementary Information

**Additional file 1.**

## Data Availability

No datasets were generated or analysed during the current study. This study did not generate new reagents. Information about all data that is presented or reanalyzed from other sources is available from the authors.
