# Supplementary material for: The Four Core Genotypes mouse model: evaluating the impact of a recently discovered translocation
Source: Biol Sex Differ. 2024 Oct 31;15:90. doi: 10.1186/s13293-024-00665-5 (PMC11529163; doi:10.1186/s13293-024-00665-5)
Supplement: Supplementary file 1 — Additional file 1. [file 13293_2024_665_MOESM1_ESM.pdf]

First Author	Year	DOI	Article Title	Mouse strain: MF1 FCG	Mouse strain: B6 FCG	Mouse strain: XY*	Sex Effect: Gonadal Effect	Sex Effect: Sex Chr Effect	X/Y gene: X/Y gene KO	X/Y gene: X/Y Gene implicated	Description sex differences evaluated
**MF1 FCG (and other non-C57BL/6 backgrounds) — not expected to carry translocation**											
De Vries GJ	2002	10.1523/JNEUROSCI.22-20-090	A model system for study of sex chromosome effects on sexually dimorphic neural and behavioral traits	✓	-	-	✓	✓	-		Male copulatory behavior, social exploration behavior, and sexually dimorphic neuroanatomical structures in the septum, hypothalamus, and lumbar spinal cord
Carruth LL	2002	10.1038/nn922	Sex chromosome genes directly affect brain sexual differentiation	✓	-	-	-	✓	-		Differentiation of mesencephalon and diencephalon dopaminergic neurons in mice embryos
Xu J	2002	10.1093/hmg/11.12.1409	Sex differences in sex chromosome gene expression in mouse brain	✓	-	-	-	-	-		Expression of X-Y homologous gene pairs in the brain across different ages
Ishikawa H	2003	10.1095/biolreprod.102.012641	Effects of Sex Chromosome Dosage on Placental Size in Mice	✓	-	✓	-	✓	-		The effect of imprinting on placental hyperplasia in late pregnancy
Markham JA	2003	10.1016/S0306-4522(02)00554-	Sex differences in mouse cortical thickness are independent of the complement of sex chromosomes	✓	-	-	✓	-	-		Cerebral cortex morphology
Durcova-Hills G	2004	10.1016/j.ydbio.2003.12.018	Analysis of sex differences in EGC imprinting	✓	-	-	-	✓	-		Methylation patterns of H19 and Igf2
Wagner CK	2004	10.1210/en.2003-1219	Neonatal mice possessing an Sry transgene show a masculinized pattern of progesterone receptor expression in the brain independent of sex chromosome status	✓	-	-	✓	-	-		Expression of progesterone receptor in forebrain of neonatal mice
Palaszynski KM	2005	10.1210/en.2005-0284	A yin-yang effect between sex chromosome complement and sex hormones on the immune response	✓*	-	-	✓	✓	-		Immune cell response to autoantigen including proliferation and cytokine production
Xu J	2005	10.1002/jnr.20429	Spatially and temporally specific expression in mouse hippocampus of Usp9x, a ubiquitin-specific protease involved in synaptic development	✓	-	-	-	✓	-		Spatial and temporal analysis of Usp9x expression in mouse hippocampus
Xu J	2005	10.1080/07435800500229243	Sexually dimorphic expression of co-repressor Sin3A in mouse kidneys	✓	-	-	✓	-	-		Expression of *Sin3a* in the kidney
Xu J	2005	10.1111/j.1460-9568.2005.0413	Sexually dimorphic expression of Usp9x is related to sex chromosome complement in adult mouse brain	✓	-	-	-	✓	-		*Usp9x* expression in the brain
Xu J	2006	10.1016/j.modgep.2005.06.011	Sexually dimorphic expression of the X-linked gene Eif2s3x mRNA but not protein in mouse brain	✓	-	-	-	✓	-	*Eif2s3x*	*Eif2s3x* expression and protein levels in the brain
Quinn JJ	2007	10.1038/nn1994	Sex chromosome complement regulates habit formation	✓	-	-	-	✓	-		Food-reinforced instrumental habit formation
Gioiosa L	2008	10.1016/j.jpain.2008.06.001	Sex chromosome complement affects nociception and analgesia in newborn mice	✓	-	-	✓	✓	-		Thermal nocioception, response to acute effects of morphine and k-opiate
Park JH	2008	10.1111/j.1601-183X.2008.0039	Effects of sex chromosome aneuploidy on male sexual behavior	✓	-	-	-	✓	-		The effect of sex chromosome aneuploidy on male sexual behaivor.
Smith-Bouvier DL	2008	10.1084/jem.20070850	A role for sex chromosome complement in the female bias in autoimmune disease	✓*	✓	-			-		Susceptibility to two immune related diseases: experimental autoimmune encephalomyelitis (EAE) and pristane-induced lupus
Xu J	2008	10.1523/JNEUROSCI.5382-07.2	Sex-specific differences in expression of histone demethylases Utx and Uty in mouse brain and neurons	✓	-	-	-	✓	-	*Utx Uty*	Comparison of *Uty* and *Utx* expression in the brain
Xu J	2008	10.1371/journal.pone.0002553	Sex-specific expression of the X-linked histone demethylase gene Jarid1c in brain	✓	-	-	-	✓	-		Expression of *Jarid1c* (*Kdm5c*) in the brain
Chen X	2009	10.1111/j.1460-9568.2009.0661	X chromosome number causes sex differences in gene expression in adult mouse striatum	✓	-	-	-	✓	-		Nigostriatial system, mRNA expression of *Pdyn, Tac1,* and *Drd2*
Barker JM	2010	10.1523/JNEUROSCI.0548-10.2	Dissociation of genetic and hormonal influences on sex differences in alcoholism-related behaviors	✓	-	-	-	✓	-		Alcohol-habit formation
Ji H	2010	10.1161/HYPERTENSIONAHA.1	Sex chromosome effects unmasked in angiotensin II-induced hypertension	✓	-	-	-	✓	-		Differences in mean arterial pressure in hypertensive model
Liu J	2010	10.1186/2042-6410-1-6	Sex differences in renal angiotensin converting enzyme 2 (ACE2) activity are 17β-oestradiol-dependent and sex chromosome-independent	✓	-	-	✓	-	-		Renal ACE2 activity
Caeiro XE	2011	10.1161/HYPERTENSIONAHA.1	Sex chromosome complement contributes to sex differences in bradycardic baroreflex response	✓	-	-	-	✓	-		Angiotenisin II bradycardic baroreflex
Cox KH	2011	10.1111/j.1601-183X.2011.0068	Sex differences in juvenile mouse social behavior are influenced by sex chromosomes and social context	✓	-	-	-	-	-		Social interactions and nonsocial behaviors
Sasidhar MV	2012	10.1136/annrheumdis-2011-201	The XX sex chromosome complement in mice is associated with increased spontaneous lupus compared with XY	✓*	-	-	-	✓	-		Lupus survial rate, kidney pathology, and gene expression in immune cells from spleen
Chen X	2013	10.1210/en.2012-2098	X and Y chromosome complement influence adiposity and metabolism in mice	✓	-	✓	✓	✓	-		Body weight, adiposity, and energy metabolism

Kopsida E	2013	10.1371/journal.pone.0073699	Dissociable effects of Sry and sex chromosome complement on activity, feeding and anxiety-related behaviours in mice	✓	-	-	✓	✓	-		Effects on activity, feeding, and anxiety-related behaivors
Dadam FM	2014	10.1152/ajpregu.00447.2013	Effect of sex chromosome complement on sodium appetite and Fos-immunoreactivity induced by sodium depletion	✓	-	-	✓	✓	-		Effect of SCC on Sodium depletion- induced brain FOS-ir and sodium and water intake induced by furo/LSD treatment; fluid and blood pressure homeostasis
Scerbo MJ	2014	10.3389/fncel.2014.00188	Neurogenin 3 mediates sex chromosome effects on the generation of sex differences in hypothalamic neuronal development	✓	-	-	-	✓	-		Neuritogenesis and gene expression in hypothalamic neurons from embryonic mice
Cisternas CD	2015	10.1016/j.mce.2015.07.027	Sex chromosome complement determines sex differences in aromatase expression and regulation in the stria terminalis and anterior amygdala of the developing mouse brain	✓	-	-	-	✓	-		Aromatase expression in stria terminalis and anterior amygdala in mice embryos
Cisternas CD	2017	10.1038/s41598-017-05658-6	Regulation of aromatase expression in the anterior amygdala of the developing mouse brain depends on ERβ and sex chromosome complement	✓	-	-	-	✓	-		Sex differentiation of amygdala in mice embryos (aromatase regulation)
Dadam FM	2017	10.1016/j.mce.2017.02.041	Sex chromosome complement involvement in angiotensin receptor sexual dimorphism	✓	-	-	-	✓	-		Relative gene expression of basal Agtr1a, Agtr2, and Mas1 receptors at fore/hindbrain nuclei and at medulla/cortical kidney
Golden LC	2019	10.1073/pnas.1910072116	Parent-of-origin differences in DNA methylation of X chromosome genes in T lymphocytes	✓*	✓	✓	-	✓	-		X chromosome DNA methylation imprinting in T cells
Cisternas CD	2020	10.1038/s41598-020-65183-x	Estradiol-dependent axogenesis and Ngn3 expression are determined by XY sex chromosome complement in hypothalamic neurons	✓	-	-	-	✓	-		Neuritogenesis in hypothalamic neurons
Cabrera Zapata LE	2021	10.1007/s00018-021-03945-0	X-linked histone H3K27 demethylase Kdm6a regulates sexually dimorphic differentiation of hypothalamic neurons	✓	-	-	-	✓	-	*Kdm6a*	Differentiation of hypthalamic neurons. Used chemical inhibition and siRNA knockdown of a specific X chromosome gene
Bautista-Abad A	2024	10.1007/s11357-024-01268-z	Aging is associated with sex-specific alteration in the expression of genes encoding for neuroestradiol synthesis and signaling proteins in the mouse trigeminal somatosensory input	✓			✓				Sex differences in expression of aromatase, estrogen and androgen receptors in the trigeminal ganglion
**C57BL/6 FCG studies with only gonadal sex effects* — unlikely impact of translocation**													
Broestl L	2022	10.1038/s42003-022-03743-9	Gonadal sex patterns p21-induced cellular senescence in mouse and human glioblastoma	-	✓	-	✓	-	-		Role of p21 in senesence induction in response to irradidation
Dhakal S	2024	10.1128/mbio.00326-24	Estradiol mediates greater germinal center responses to influenza vaccination in female than male mice	-	✓	-	✓	-	-		Response to inactivated influenza vaccine with effect on B cells, durability of immunity, and protection from infection
Dill-Garlow R	2019	10.1038/s41598-018-37175-5	Sex Diferences in Mouse Popliteal Lymph Nodes	-	✓	-	✓	-	-		Delayed-type hypersensitivity (DTH) response
Ghosh MK	2021	10.3389/fendo.2021.582614	Sex Differences in the Immune System Become Evident in the Perinatal Period in the Four Core Genotypes Mouse	-	✓	-	✓	-	-		Number of immune cells in thymus and spleen
Kuo J	2010	10.1186/2042-6410-1-7	Sex differences in hypothalamic astrocyte response to estradiol stimulation	-	✓	-	✓	-	-		Hypothalamic astrocytes differentiation and response to estradiol stimulation
Manwani B	2015	10.1038/jcbfm.2014.186	Sex differences in ischemic stroke sensitivity are influenced by gonadal hormones, not by sex chromosome complement	-	✓	-	✓	-	-		Ischemic stroke effect using middle cerebral artery occlusion (MCAO) model and evaluated infarct volume
Sakamuri A	2023	10.1530/mah-23-0010	Sex hormones, sex chromosomes, and microbiota: Identification of *Akkermansia muciniphila* as an estrogen-responsive microbiota.	-	✓	-	✓	-	-		Effect on microbiota compoistion in the gut
**C57BL/6 FCG combined with XY* and/or specific X/Y gene candidates — unlikely impact of translocation**													
Alsiraj Y	2017<br>2021	10.1161/CIRCULATIONAHA.116.023789<br>10.1161/ATVBAHA.120.314407	Female mice with an XY sex chromosome complement develop severe angiotensin II-induced abdominal aortic aneurysms<br>Monosomy X in female mice influences the regional formation and augments the severity of angiotensin II-induced aortopathies		✓*	✓		✓			Sex chromosome dosage and sex differences in pathology of aortic aneurysm
Chen X	2008	10.1002/dneu.20581	Sex difference in neural tube defects in p53-null mice is caused by differences in the complement of X not Y genes	-	✓	✓	-	✓	-		Neural tube defects in *p53*-null mice
Wijchers PJ	2010	10.1016/j.devcel.2010.08.005	Sexual dimorphism in mammalian autosomal gene regulation is determined not only by Sry but by sex chromosome complement as well	-	✓*	✓*	✓	✓	-		Sex effects on regulation of gene expression in thymus
Bonthuis PJ	2012	10.1016/j.yhbeh.2012.02.003	X-chromosome dosage affects male sexual behavior	-	✓	✓	✓	✓	-		Sexual behavior in mice such as ejaculation, thrusting, and mounting
Chen X	2012	10.1371/journal.pgen.1002709	The number of X chromosomes causes sex differences in adiposity in mice	-	✓	✓	✓	✓	-		Role of sex chromosome dosage in body weight, adiposity, and energy metabolism
Li J	2014	10.1093/cvr/cvu064	The number of X chromosomes influences protection from cardiac ischaemia/reperfusion injury in mice: one X is better than two	-	✓	✓	-	✓	-		Myocardial ischaemia reperfusion injury
Link JC	2015	10.1161/ATVBAHA.115.305460	Increased high-density lipoprotein cholesterol levels in mice with XX versus XY sex chromosomes	-	✓	✓	✓	✓	-		Circulating plasma lipid levels and lipoprotein profile.
Kaneko S	2018	10.1126/sciadv.aar5598	X chromosome protects against bladder cancer in females via a KDM6A-dependent epigenetic mechanism	-	✓	-	✓	✓	✓	*Kdm6a*	Effect on bladder cancer development and survival

Itoh Y	2019	10.1172/JCI126250	The X-linked histone demethylase Kdm6a in CD4+ T lymphocytes modulates autoimmunity	√*	√	-		√	√	*Kdm6a*	Role of T cells in experimental autoimmune encephalomyelitis including T cell subtypes, activation, proliferation, and cytokine production.
Link JC	2020	10.1172/JCI140223	X chromosome dosage of histone demethylase KDM5C determines sex differences in adiposity		√			√	√	*Kdm5c*	Sex chromosome and Kdm5c gene dosage effects on adiposity, energy metabolism, and adipocyte differentiation
Qi S	2021	10.1186/s12974-021-02120-3	X chromosome escapee genes are involved in ischemic sexual dimorphism through epigenetic modification of inflammatory signals	-	√	-	-	√	-	*Kdm5c*<br>*Kdm6a*	Ischemic stroke: gene expression and cytokine response of microglia from mice with middle cerebral artery occlusion (MCAO)
Shi W	2021	10.1016/j.devcel.2021.09.022	Cardiac proteomics reveals sex chromosome-dependent differences between males and females that arise prior to gonad formation	-	√	√	√	√	-		Transcriptomic and proteomic analysis of hearts
Taylor AMW	2022	10.1002/jnr.24704	Sex differences in kappa opioid receptor antinociception is influenced by the number of X chromosomes in mouse	-	√	√	-	√	-		Analgesic response via kappa opioid receptor antinociception
Zhang P	2022	10.1038/s41467-024-49764-2	X chromosome dosage drives statin-induced dysglycemia and mitochondrial dysfunction	-	√	-	√	√	√	*Kdm5c*	Response to statin treatment related to dysglycemia and mitochondrial function
Cheng MI	2023	10.1038/s41590-023-01463-8	The X-linked epigenetic regulator UTX controls NK cell-intrinsic sex differences	-	√	-	-	√	√	*Kdm6a*	NK cell UTX expression levels, *Kdm6a*
Lopez-Lee C	2023	10.1101/2023.09.19.558439	Sex Chromosomes and Gonads Shape the Sex-Biased Transcriptomic Landscape in Tlr7-Mediated Demyelination During Aging	-	√	-	√	√	√	*Trl7*	Evaluated oligodendrocyte and microglial responses to demyelination

*Does not exclude the possibility of gonad–sex chromosome interaction, which was not tested in all publications
